# Two Different Strategies to Enhance Osseointegration in Porous Titanium: Inorganic Thermo-Chemical Treatment Versus Organic Coating by Peptide Adsorption

**DOI:** 10.3390/ijms19092574

**Published:** 2018-08-30

**Authors:** Monica Ortiz-Hernandez, Katrin S. Rappe, Meritxell Molmeneu, Carles Mas-Moruno, Jordi Guillem-Marti, Miquel Punset, Cristina Caparros, Jose Calero, Jordi Franch, Mariano Fernandez-Fairen, Javier Gil

**Affiliations:** 1 Biomaterials, Biomechanics and Tissue Engineering Group (BBT), Department of Materials Science and Metallurgical Engineering, Universitat Politècnica de Catalunya (UPC), 08019 Barcelona, Spain; Monica.ortiz-hernandez@upc.edu (M.O.-H.); meritxell.molmeneu@upc.edu (M.M.); carles.mas-moruno@upc.edu (C.M.-M.); jordi.guillem.marti@upc.edu (J.G.-M.); miquel.punset@upc.edu (M.P.); Cristina.caparros@upc.edu (C.C.); jose.calero@upc.edu (J.C.); 2 Barcelona Research Center in Multiscale Science and Engineering, Universitat Politècnica de Catalunya (UPC), 08019 Barcelona, Spain; 3 Departamento de Cirugía Animal, Facultad de Veterinaria, Universidad Autónoma de Barcelona, Bellaterra, 08193 Barcelona, Spain; Katrin.Rappe@uab.cat (K.S.R.); Jordi.franch@uab.cat (J.F.); 4 Facultad de Odontología, Campus de Medicina y Ciencias de la Salud, Universidad Internacional de Cataluña (UIC), 08017 Barcelona, Spain; mferfai@gmail.com

**Keywords:** titanium foams, osseointegration, porosity, bioactive materials

## Abstract

In this study, highly-interconnected porous titanium implants were produced by powder sintering with different porous diameters and open interconnectivity. The actual foams were produced using high cost technologies: Chemical Vapor Deposition (CVD), Physical Vapor Deposition (PVD), and spark plasma sintering, and the porosity and/or interconnection was not optimized. The aim was to generate a bioactive surface on foams using two different strategies, based on inorganic thermo-chemical treatment and organic coating by peptide adsorption, to enhance osseointegration. Porosity was produced using NaCl as a space holder and polyethyleneglicol as a binder phase. Static and fatigue tests were performed in order to determine mechanical behaviors. Surface bioactivation was performed using a thermo-chemical treatment or by chemical adsorption with peptides. Osteoblast-like cells were cultured and cytotoxicity was measured. Bioactivated scaffolds and a control were implanted in the tibiae of rabbits. Histomorphometric evaluation was performed at 4 weeks after implantation. Interconnected porosity was 53% with an average diameter of 210 µm and an elastic modulus of around 1 GPa with good mechanical properties. The samples presented cell survival values close to 100% of viability. Newly formed bone was observed inside macropores, through interconnected porosity, and on the implant surface. Successful bone colonization of inner structure (40%) suggested good osteoconductive capability of the implant. Bioactivated foams showed better results than non-treated ones, suggesting both bioactivation strategies induce osteointegration capability.

## 1. Introduction

Titanium (Ti) and its alloys have been widely used as constitutive material for dental and orthopedic implants due to their excellent corrosion resistance and biocompatibility, and the possibilities of direct contact between implant and bone [1,2]. Moreover, Ti-based porous scaffolds have been used to fix implants to bone through bone ingrowth into the porous system, and even as a bone substitute. In this regard, three-dimensionally interconnected pores allow an easy and fast penetration of bone-forming cells, and attachment and proliferation of vascularized new bone, thus providing a strong and durable implant–bone interaction [3]. The surface roughness of the porous structure gives immediate primary mechanical stability to the implant due to the high friction forces between the metal implant and peri-implant bone. Furthermore, the porosity of the system reduces the implant’s Young’s modulus, improving load transfer to adjacent bone and avoiding deleterious stress shielding and bone resorption [4].

A wide range of processes have been reported regarding the manufacture porous titanium implants, such as polymeric sponge replication [5], compression and sintering [6], combustion synthesis [7], selective electron beam melting [8,9], rapid prototyping [10,11], powder metallurgy (PM) [11,12,13,14], selective laser melting [15,16], and selective laser sintering [14,17]. PM seems to be a particularly advantageous method for manufacturing complex shapes with interconnected pores without the need for machining steps [13,18] given its processing route and cost [13,18,19]. In PM, pores can be originated from the particle arrangement when compacting or from changes in this arrangement as spacer particles disintegrate, and from solid-state diffusion during the sintering step [20]. Pore size, porosity, pore distribution, and interconnectivity can be well optimized using this technique.

Ti can also undergo surface modifications to improve cell adhesion and osseointegration [21,22,23], and the surface of the porous system can be activated using different methods to potentiate osseointegrative properties. The substrate material can be coated with nonstructural materials such as calcium phosphates [24,25,26], demineralized bone matrix [26], bone marrow aspirate [27], platelet-rich plasma [28], bone morphogenetic protein [29,30], mesenchymal stem cell [31,32], different types of bioactive peptides [33,34], among others. The application of silanes has been studied and several molecules are applied in biomedical applications [35,36]. However, effectiveness and safety of these types of modification are not totally well-documented to date [37].

In this study, a highly-interconnected porous Ti scaffold obtained by PM, was treated using two different biomimetic methods of surface activation:

(1) thermo-chemical treatment, which promotes the nucleation and growth of a bone-like apatite layer over the Ti surface [38,39,40,41,42];
(2) grafting of an arginine-glycine-aspartic acid (RGD) cell adhesive peptide, derived from a bone extracellular matrix (ECM) [43,44,45,46] on the surface of the scaffold. ECM-derived molecules, such as RGD peptides, are capable of interacting with cell-expressed receptors like integrins, which trigger the biological processes required for an optimal osseointegration [33].

The hypothesis of this study was to compare the osseointegration of the Ti porous foam between two strategies of surface activation: an inorganic thermo-chemical treatment with the grafting bioactive peptides on the surface of the scaffold.

The combination of sintered interconnected porous titanium with treatments of bioactivation represents an excellent strategy to improve the osseointegration of vertebrae implants, and orthopedic and maxillofacial prosthesis.

## 2. Results

### 2.1. Structure of Porous System

Table 1 shows the characteristics of the porous titanium structure obtained, the interconnected porosity (I) of the scaffolds was 53% with macropores of 210 µm of diameter on average (P). Bioactivation of the scaffolds did not significantly modify such values of interconnectivity (Ti activated thermo-chemically, 57%; Ti activated using peptides, 56%), showing no statistically significant differences between treated and untreated samples in terms of pore size and/or interconnectivity. The microroughness obtained by the grit-blasting process with alumina particles produced a roughness of around 1 μm. The bioactive treatments do not affect microroughness.

The thermo-chemical treatment for bioactivation did not modify the porosity of the sintered porous Ti samples, consistent with results of Mercury Intrusion Porosimeter (MIP) tests, because the nanometric scale of coating thickness formed in both strategies, although it would modify the surface composition, and the specific surface and the degree of oxidation, among the most outstanding structural aspects [41,42]. At the structural level, the bioactivation treatment by RGD would only cause changes at the level of chemical surface composition [43,44,45,46]. The same occurred with the microroughness of the foams [38,44].

### 2.2. Mechanical Properties

The porous Ti implants produced in this study showed adequate mechanical properties and a Young’s modulus (E) close to that of the cancellous bone (Table 2). Compression tests for the Ti porous material revealed a yield strength of about 105 MPa with a maximum strength of 170 MPa, and a strain up to fracture of 30%. It can be observed that the mechanical properties (σ_0_ and σ_max_) of titanium porous implants with thermo-chemical and peptides adhesion treatments slightly increased, while strain to fracture decreased. This phenomenon was ascribed to the incorporation of oxygen into the structure producing these small variations. These values are higher than NiTi or Ta porous structures and other highly interconnected biomaterials. Differences are mainly associated to the nature of the materials as well as the processing method. The manufacturing process of porous tantalum used chemical vapor deposition/infiltration (CVD/CVI) to create a porous metal construct. Cylinders of porous NiTi were produced by self-propagating high temperature synthesis (SHS).

The fatigue behavior of the studied titanium porous implants indicated that the fatigue limit at 10^8^ cycles was higher than the values of the tantalum foams and the NiTi foams, as can be observed in Table 2, therefore showing excellent fatigue results. These results demonstrate that the mechanical properties of porous titanium obtained by the present process are adequate for biomedical applications, such as vertebral prosthesis. Ta prostheses are currently applied as spine spacers in humans.

### 2.3. In Vitro Characterization

**Figure 1:**
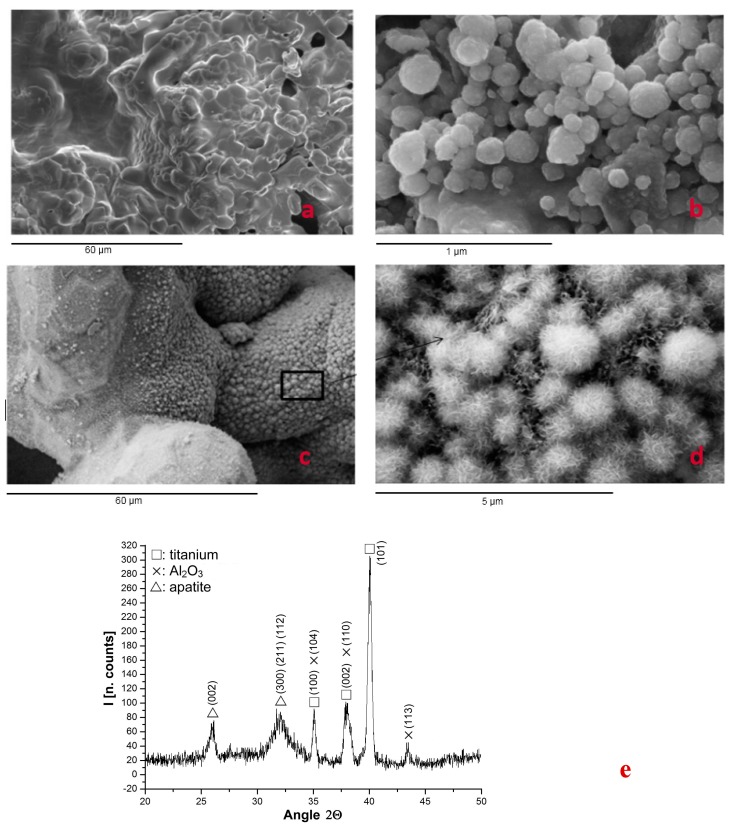
Surface images of different samples: (**a**) no activated porous Ti; (**b**) Bioglass control sample; (**c**) cross-section of a porous sample with thermo-chemical treatment after its immersion in SBF, obtained after the fracture of the specimen under cryogenic conditions showing apatite on the surface of porous titanium foam; (**d**) magnification of the preceding image; (**e**) X-ray pattern of the layer deposited on the surface with peaks corresponding to the apatite. Ti oxides are not usually observed, since the thickness of the Ti oxide layer is less than 4 nm and it is completely coated with apatite of hundreds of μm. Alumina particles have sizes from 40 to 100 μm and can be detected using the X-Ray Diffraction (XRD).

Thermochemically treated samples were observed using Scanning Electron Microsocopy (SEM) after 10 days in a simulated body fluid (SBF) immersion to analyze the presence of apatite. SEM images and an X-ray diffractogram showed apatite over the implant and inside the porous structure, as well as over the Bioglass control (Figure 1). The presence of crystalline apatite over the Bioglass control validated the proper execution of the bioactivity test, and on the other hand, apatite over the inner surfaces of porous titanium samples reflect the bioactivity of the treatment. The peaks of Al_2_O_3_ were due to the residual alumina particles on the titanium surface produced by the mechanical anchorage of the abrasive particles with the titanium. The residual content was lower than 1%. These particles are bioinert and they have no influence over the biological response [41,42].

**Figure 2:**
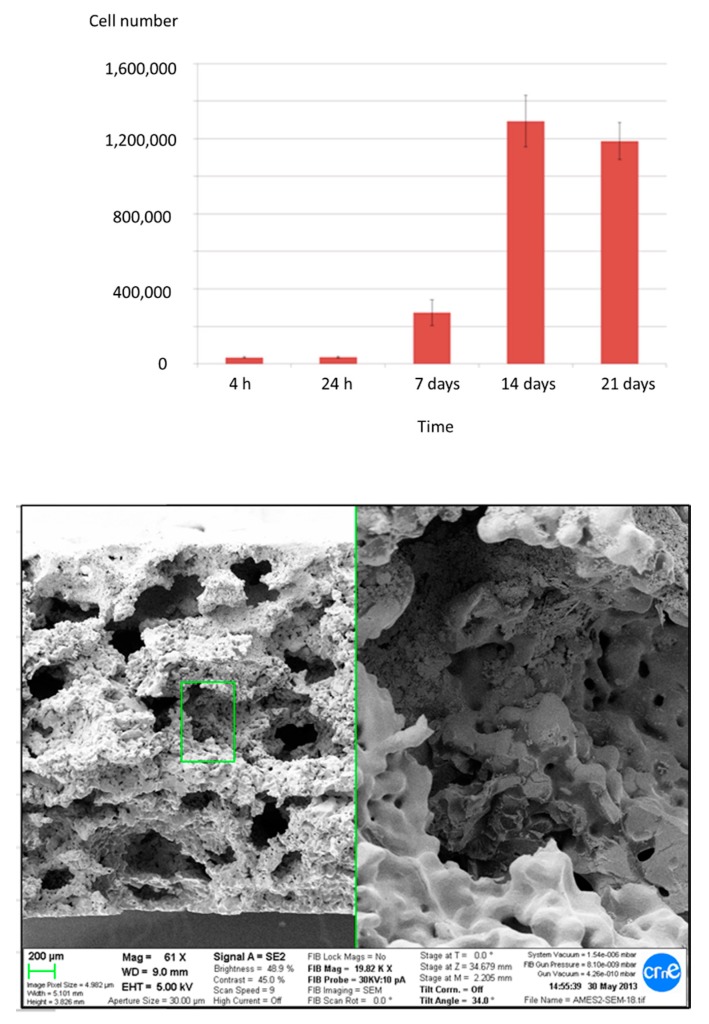
Proliferation of osteoblastic human cells results with the detail of the SEM micrographs showing cell colonization of the inner core of porous bioactivated titanium samples.

Cells proliferated adequately on the porous samples until they reached confluence at 14 days (Figure 2). In order to evaluate the cell colonization inside the porous titanium, cross-sections of the material were studied at each cell-proliferation time-point. At initial culture times (4 and 24 h), cells mainly occupied the surface of the samples without penetrating the porous structure. After 7 days of cell culture, cells started to enter the pores, infiltrating more at 14 days. What is noteworthy, after 21 days, cells proliferated inside inner pores almost filling them, as can be seen in the SEM micrograph of Figure 2 showing cells inside the pores and interconnections of the core inner part of the porous bioactivated samples.

**Figure 3:**
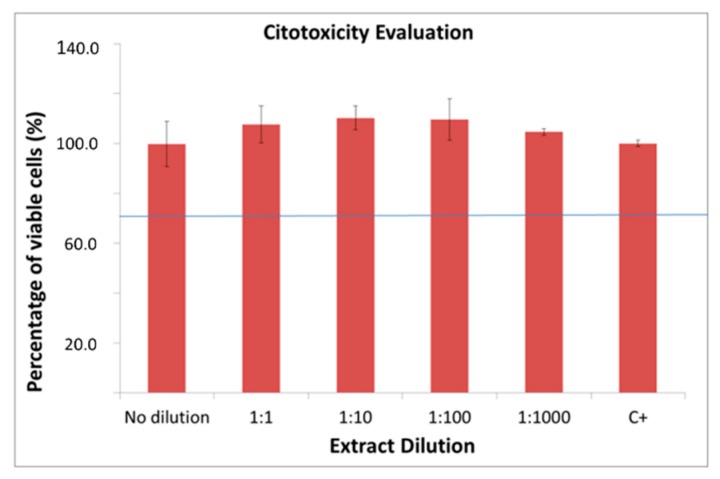
Viability of the cells in the biocompatibility test on porous samples of titanium with thermo-chemical treatment.

No cytotoxic effect was observed when analyzing the treated samples by measuring the Lactate Dehydrogenase (LDH) activity of osteoblastic Saos-2 cells. Cell survival rates were close to 100% of viability (Figure 3).

### 2.4. Osseointegration In Vivo

All rabbits remained in good health for the whole duration of the follow-up with no evidence of inflammation or infection in the surgical site. During implant retrieval, no clinical signs of infection or adverse tissue reaction were observed around the surgical site. In total, 36 implants were harvested and considered for further analysis.

**Figure 4:**
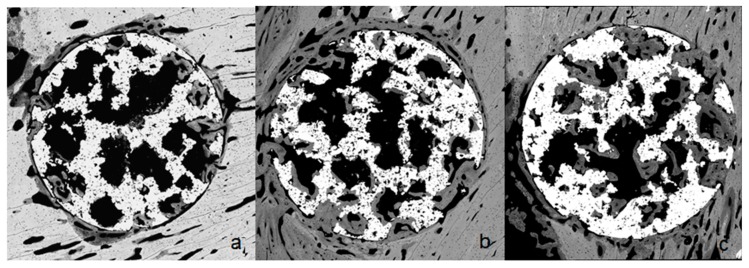
Histology of the porous titanium 14 days after implantation; (**a**) without bioactive treatment; (**b**) with thermochemical treatment; (**c**) functionalized with peptide. Diameter of the samples was 3 mm.

Initial new bone formation was observed one week after implantation. After four weeks of implantation, all types of implants supported bone formation in the inner pores (Figure 4).

**Figure 5:**
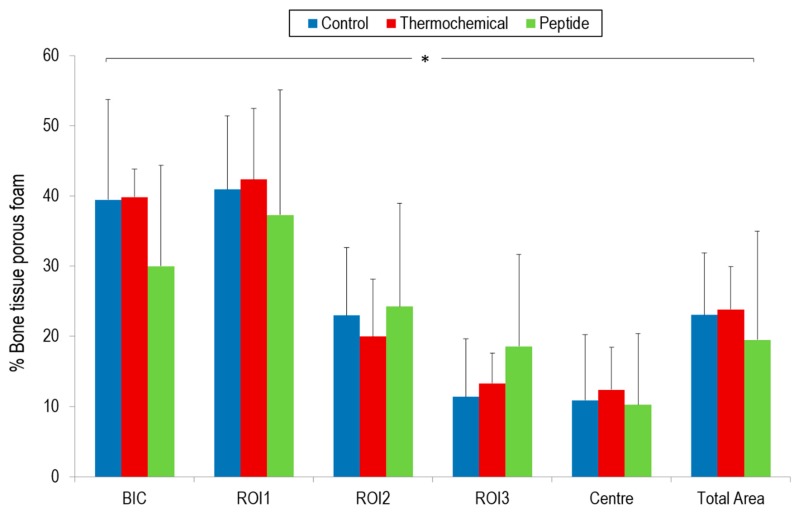
New bone formation in titanium porous foam at 4 weeks after implantation. (*) means there were no statistically significant differences (*p* > 0.05) depending on the type of samples for all analyzed parameters.

Bone Implant Contact (BIC) and new bone formed in region of interest (ROI) results in the different areas are shown in Figure 5. Control and thermo-chemically treated samples presented the highest BIC values (39.41% and 39.81%, respectively). Samples biofunctionalized with the peptide yielded the lowest BIC values.

With regard to ROI’s values, in general, non-treated samples showed a trend towards a reduced percentage of bone formed, although no statistically significant differences were observed with bioactivated samples. Thermo-chemically treated samples presented a trend of the highest ROI value in the most external quantified area (ROI1 = 42.33%). Nevertheless, peptide-treated samples showed a tendency of better ROI values for ROI2 and ROI3 inner areas (24.21% and 18.52%, respectively).

## 3. Discussion

The most common cause of bone implant failures is an impaired implant fixation and stability as a result of a poor osseointegration (i.e., insufficient bone ongrowth or ingrowth). Another relevant problem is the resorption and bone remodeling of surrounding bone through the stress-shielding effect induced by the stiffness of the implant [51,52,53,54]. An improved biological fixation can be attained by modifying the structure of the bulk material and combining micro-nano physico-chemical features on the surface of the constitutive material. Such a strategy would overcome both the two main disadvantages of metallic biomaterials: their rigidity and lack of biological recognition. To this end, in this work we have produced porous scaffolds that have been bioactivated at the surface level.

Porous implants are able to achieve optimal levels of biological fixation, while at the same time reducing the elastic modulus and relieving the stress-shielding phenomena [53,54,55,56,57]. For instance, introducing porosity to commercially pure titanium, the modulus of elasticity could be reduced from 110 GPa to less than that of the human cancellous bone [54,56,57]. Nonetheless, a certain balance must be maintained between the lessening of the Young’s modulus and the strength reduction when increasing the implant porosity [3].

Fatigue tests that exceeded 10^8^ cycles were always performed below the elastic limit, which explains why the samples tested retained the same initial porosity and structure, and why no statistically significant changes were observed in their elastic modulus. In the last three cycles before the fracture, a plastic deformation and a decrease in the porosity due to the collapse of the pores were observed, with an approximate increase in the elasticity modulus of 10%. However, this material in its usual applications, being always in the elastic regime, will not suffer changes in the modulus of elasticity.

PM has proved to be an effective method to produce porous scaffolds with an open-cell structure and interconnected pores for biomedical applications, allowing an adequate control of pore size and porosity to promote bone ingrowth [11,13]. This method creates macro- and micropores, facilitating bone ingrowth, vascularization, and bodily fluid flow throughout [51,52]. The pore dimensions are determined by the size of the space holders and titanium powder particles respectively [53]. Sieved NaCl particles were selected as a space holder agent [54,55] in our study due to their ability to completely decompose at low temperatures, avoiding reactions with the titanium powder and the formation of impurities within the foam [56].

The pore size and porosity in our study were within the ranges considered optimal in the literature, approximately 50% of porosity with pores between 100 and 400 μm [13,21,51,54,57,58,59,60,61,62], chosen as a compromise between capability and the rate of bone ingrowth [3,63], while maintaining the mechanical strength of the porous material [11,18,20,64,65]. Our porous scaffolds exhibited adequate static and dynamic mechanical properties for clinical use under load-bearing conditions, and excellent biomechanical compatibility with the Young’s modulus more similar to that of the cancellous bone than other metallic foams, such as NiTi and tantalum [66,67,68], which have also been widely used in orthopedic applications.

All the materials used in this study have shown to be perfectly biocompatible with osteoblast-like cells. The treated samples with the thermo-chemical method did not display any cytotoxic effect. Ti and Ti functionalized with RGD were devoid of toxicity for osteoblastic cells. This has been proved in a large number of previous studies. In particular, we have demonstrated that functionalizing Ti surfaces with RGD peptides using silanization showed no toxicity for cells and supports excellent proliferation rates for osteoblasts and mesenchymal stem cells [43,46].

In order to enhance cellular adhesion and organization within the porous scaffold, we have used the thermo-chemical treatment method, with a well-demonstrated capacity to improve osteoconductivity as well as osteoinductivity [39,62]. Alternatively, we have also used RGD peptides to integrate cell-recognizable ligands and signaling on the surface of the scaffold to stimulate cell adhesion, proliferation, and differentiation. There is evidence that RGD-peptides, known as recognition motifs for several integrins, promote cellular adhesion, influence cellular proliferation, and differentiation of local cells. However, in this study, the RGD-coating did not show an advantage in comparison with the thermo-chemically biofunctionalized Ti, as it was also shown by other authors comparing RGD-coated Ti with polished and sandblasted Ti [52,59,69].

This behavior could be related to a different mechanism of interaction for each strategy. The thermo-chemical treatment generates a sodium titanate layer on the implant surface, promoting apatite formation and deposition over the implant surface, providing the mineral phase and enhancing new bone ongrowth on the external surface of implant. This fact could explain the highest trend in BIC and ROI1 observed for thermo-chemical treated samples, whereas it could be more difficult for new bone formation to get into the inner porous structure. However, bone ingrowth was also detected inside the porous scaffold, but at a lower rate. It could be argued that the thermo-chemical treatment was more efficient on the external and peripheral areas of the implant, thereby ensuring better primary implant fixation. In the inner zones of the implant, a peptide treatment could improve bone ingrowth inside the porous material more than the thermo-chemical treatment, producing better results in the internal areas in terms of bone ingrowths and vascularization (i.e., ROI2 and ROI3).

The rate of bone ingrowth into the porous specimens was in the order shown by Baril et al. [57], as can be expected using analogous experimental conditions. Our results are also similar to those reported by Vasconcellos et al., with a Ca/P ratio at the bone-implant interface and bone ingrowth remarkably enhanced at 4 weeks in the porous scaffolds of pure Ti fabricated by PM and inserted in the proximal tibia of 21 rabbits [13], and comparable to those of Ponader et al. concerning bone ingrowth in selective electron beam-melted Ti–6Al–4V porous structures implanted in the frontal skull of domestic pigs, but noticeably poorest for the bone-implant contact [9]. The surface activity of the diverse materials and the different experimental conditions may be in the base of the variations observed in the results.

Nonetheless, it should be noted that the values observed were not statistically significant and therefore these assumptions should be taken with caution.

## 4. Material and Methods

### 4.1. Scaffold Production

The porous titanium scaffold was produced using a PM technique by mixing commercially pure titanium (CP) Grade 2, with a mean grain size of about 80 μm, with NaCl particles ranging from 300 to 600 μm of diameter as a space holder, in a 65-to-35 percent volume ratio. The NaCl “space holder” particle size distribution was analyzed using laser granulometry by using a Beckman Coulter LS Particle Size Analyzer (Beckman Coulter Life Sciences, Indianapolis, IN, USA), with a normal distribution of particle diameter that had a mean value of 496.4 μm and a Standard Deviation (SD) of ±119.7 μm. In order to homogenize the mixture, ethylene glycol (15 wt.%) was added as a binder phase and removed at 200 °C in air.

The mixture was uniaxially pressed under 100 MPa in a stainless-steel mold and then isostatically pressed under 200 MPa. The space holder was removed by washing samples several times with distilled water, ensuring the total removal of NaCl by controlling the electrical conductivity of the rinsing water until conductivity remained stable. Sintering of porous titanium was carried out at 1350 °C for 2 h under high vacuum conditions at 5 × 10^−4^ mbar. Characterization of micro- and macroporosity size (pore diameters greater than 10 μm) and evaluation of porosity interconnections were done using mercury immersion porosimetry (MIP) by using AutoPore IV 9500 V1.07 equipment (MIP, Micrometrics, Norcross, GA, USA) [36]. MIP tests used a 3 cm^−3^ penetrometer and tridistilled mercury (Panreac, Barcelona, Spain). An intrusion pressure series was analyzed between 0.0034 MPa (0.5 psia) and 206.84 MPa (30,000 psia).

Implants with microestructured surfaces have been reported to have a more intensive bone implant contact than implants with smooth machined surfaces, resulting in higher mechanical retention when implanted in humans [37,38]. For this aspect, all samples were treated with alumina particles (90–120 µm in size) with a 0.25 MPa blasting pressure until achieving a roughness saturation. Surface profiles were measured with a contact 2-D profilometer (Surftest SV500^©^, Mitutoyo, Neuss, Germany), and roughness profiles were calculated by filtering the surface profiles with a Gaussian filter. A 0.8-mm “cut-off” value was applied for filtering. The Ra (arithmetic average of peak-valley height) and Pc (number of falling flanks in a given distance) were calculated from the roughness profiles with appropriate software (SurfpackTM v3.00, Mitutoyo, Japan), according to international standards (ISO4287:1997).

The sintered cylindrical samples used for in vivo implantation presented an average diameter of 3 mm and a length of 6 mm. Cylindrical samples used for mechanical characterization and cell assay presented an average diameter of 10 mm, and a length of 15 mm according to the ISO 13314 standard and 2 mm of thickness, respectively. Six cylinders were embedded in acrylic resin and sectioned radially with a low-speed diamond-disc cutter (EXAKT 310 CL; EXAKT Advanced Technologies GmbH, Norderstedt, Germany) and polished lightly using increasingly fine sandpapers (600, 800, and 1200 grit). Metallographic examination was done using optical microscopy (Olympus 300, Tokyo, Japan) and scanning electron microscopy (JEOL 6400; JEOL USA Inc., Peabody, MA, USA) on different sections of these specimens.

### 4.2. Surface Bioactivation

Surface bioactivation was achieved by either one of two methods.

#### 4.2.1. Thermo-Chemical Treatment

The thermo-chemical procedure consisted of samples being immersed in 5 M NaOH at 60 °C for 24 h, followed by drying at 60 °C for 24 h. A 600 °C heating was performed in an ending step for 1 h [36,39,40,41,42]. This method created the appearance of a microporous layer made up of an alkaline titanate hydrogel formed during the alkaline/heat treatment. This surface layer is an amorphous sodium titanate layer containing small amounts of a mixture of crystalline sodium titanates (Na_2_Ti_5_O_11_) and rutile (TiO_2_). During the alkali treatment, the surface passive TiO_2_ layer partially dissolved into an alkaline solution because of the corrosive attack of hydroxyl groups [41,42].

TiO_2_ + OH^−^ → HTiO^3−^

This reaction is assumed to proceed simultaneously with the following hydration of the Ti metal [40,41,42].

Ti + 3OH^−^ → Ti(OH)^3+^ + 4e^−^

Ti(OH)^3+^ + e^−^ → TiO_2_·H_2_O + 0.5 H_2_ (g)

Ti(OH)^3+^ + OH^−^ ⇔ Ti(OH)_4_

A further hydroxyl attack to hydrated TiO_2_ produced negatively charged hydrates on the surfaces of the substrates as follows:

TiO_2_·nH_2_O + OH^−^ ⇔ HTiO^3−^·nH_2_O

These negatively charges species were combined with alkali ions in the aqueous solution, resulting in the formation of an alkali titanate layer. During heat treatment, the hydrogel layer was dehydrated and densified the titanate layer.

When exposed to simulated body fluid, the alkali titanate layer was again hydrated to transform into TiO_2_ hydrogel via the release of alkali ions from the alkali titanate layer in SBF. The alkali release and the ion exchange with H_3_O^+^ ions in the simulated body fluid, resulting in a pH increase in the surrounding fluid. The pH increase gave rise to an increase in the ionic activity product of apatite according to the following equilibrium in simulated body fluid [41,42].

10Ca^2+^ + 6PO_4_^3−^ + 2OH^−^ ⇔ Ca_10_(PO_4_)_6_(OH)_2_

Then, the samples were evaluated using scanning electron microscopy (SEM) and energy dispersive X-Ray spectroscopy (EDS) to visualize and identify apatite on the treated surfaces [47,49,59].

The human osteoblast cultures revealed that bioactive titanium surfaces had a better cell response than positive and negative controls [39,40]. The apatite layer had a significant effect on the adhesion (cell count) and differentiation (osteocalcine concentration) of osteoblast-like cells [39,40,41,42].

#### 4.2.2. Peptide Adhesion

**Figure 6:**
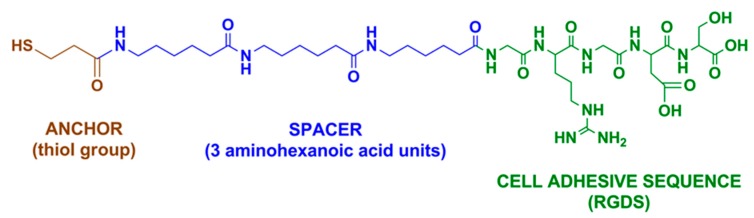
Chemical structure of the cell adhesive RGD peptide used to functionalize Ti foams.

Alternatively, bioactivation of the metallic foams was achieved using covalent grafting of an RGD cell-adhesive peptide. This peptide (Figure 6) is comprised of the sequence Gly-Arg-Gly-Asp-Ser (GRGDS) as a cell-binding motif, and three units of 6-aminohexanoic acid (Ahx) and 3-mercaptopropionic acid (MPA) as a spacer-anchoring moiety [43,44,45,46]. This peptide was synthesized in a solid-phase as previously reported [43].

Peptide attachment was accomplished by means of silanization. To this end, surfaces were initially cleaned and passivated with 65% (*v*/*v*) HNO_3_ for 1 h at room temperature. After this time, samples were rinsed in distilled water, ethanol, and acetone, and dried under nitrogen gas. Next, samples were immersed in 2% (*v*/*v*) of (3-aminopropyl) triethoxysilane (APTES; Sigma-Aldrich, St. Louis, MO, USA) in anhydrous toluene and silanized for 1 h at 70 °C under a nitrogen atmosphere. This treatment was followed by ultrasonication for 5 min in toluene to remove non-covalently bound silanes, copious washes in water and organic solvents, and curing of the silanes at 120 °C for 5 min. To ensure a chemoselective binding of the peptide via its anchor group (a thiol functionality) to the surfaces, the silane layer was further modified by reaction with 2 mg/mL of the 3-maleimidopropionic acid *N*-hydroxysuccinimide ester (Alfa-Aesar, Karlsruhe, Germany) in *N*,*N*-dimethylformamide (DMF) for 1 h at room temperature. Finally, silanized samples were each coated with a solution of RGD peptide at 200 µM in Physiological Body Solution (PBS) (pH 6.5) overnight at room temperature. Control surfaces were only treated with a buffer. After peptide conjugation, samples were washed with PBS, sterilized with 70% (*v*/*v*) EtOH during 20 min, and dried. Such a protocol of biofunctionalization has been characterized in previous studies [41,42,43,44].

### 4.3. Mechanical Properties

Mechanical properties were evaluated using compression and fatigue tests by using a MTS Bionix 370 (MTS, Eden Prairie, MN, USA) for the titanium porous structures with and without bioactive treatments. Compression tests were done according to ISO Standard 13314 for porous metals, measuring the elastic modulus and ultimate compressive strength of the cylindrical samples. Five specimens of each material were analyzed in compression tests at a cross-head speed of 2.5 mm·min^−1^. The fatigue tests were performed in compression–compression mode, using frequencies of 15 Hz and 37 °C in a physiological medium with a maximum number of cycles of 1 10^8^ to determine the fatigue limit strength. Fatigue tests were done under a load control, introducing a limit of displacement too, at different load levels of 40%, 50%, 60%, and 80% of the ultimate compressive strength. At least two samples were evaluated at each load level to establish the fatigue curve and the load was applied as a sine wave function [48,49].

### 4.4. In Vitro Biological Characterization

In vitro biological characterization was performed by culturing SaoS-2 osteoblast-like cells (ATCC HTB 85, Manassas, VA, USA) in McCoy’s 5A Medium (Sigma-Aldrich) supplemented with 10% (*v*/*v*) fetal bovine serum (FBS), 2mM glutamine, 20 mM HEPES buffer, penicillin/streptomycin (50 U/mL and 50 µg/mL), and 1 mM sodium pyruvate (Invitrogen, Carlsbad, CA, USA). Cells were maintained in an incubator at 37 °C in a humidified atmosphere and at 5% (*v*/*v*) CO_2_. SaoS-2 cells were seeded in 48-well plates to cover the porous samples placed in each well and incubated for 21 days.

Cell proliferation was analyzed at 4 and 24 h, and 7, 14, and 21 days after cell seeding by measuring the LDH activity as described above. For this assay, 50 10^3^ cells were cultured and lysed with 300 µL of Mammalian Protein extraction reagent (M-PER) at each specified time.

Cell colonization was visualized by SEM at the same time points as the cell proliferation assay. Cells were fixed with 2.5% glutaraldehyde in a 0.1 M phosphate buffer (PB) at 4 °C for 1 h and rinsed (3 times) with PB. Then, cells were immersed in 1% osmium tetroxide solution at room temperature to increase the electron contrast. Fixed samples were dehydrated in a graded ethanol series (50%, 70%, 90%, 96%, and 100%) and completely dehydrated in hexamethyldisilazane (HDMS, Sigma-Aldrich). Dried samples were observed using a field emission scanning electron (FIB/SEM) Zeiss Neon 40 (Carl Zeiss NTS GbmH, Oberkochen, Germany) at 5 kV without any gold or graphite coating. Bioglass samples (CaO–P_2_O_5_–SiO_2_) were used as control due to their known ability to produce a precipitation of a bioactive hydroxycarbonated apatite layer when immersed in biological fluids that can bond to biological tissue [2], as accepted in many references of published scientific literature.

Cytotoxicity was measured by indirect exposition following the ISO 10993-5 standard [43]. Briefly, samples were incubated in a complete medium for 72 h. Then, 5 10^3^ cells were exposed to different dilutions (1, 1:1, 1:10, 1:100, and 1:1000 in complete medium) of the conditioned supernatant for 24 h. Cells were lysed with 100 µL of mammalian protein extraction reagent (M-PER; Thermo-Scientific, Rockford, IL, USA). Afterwards, lactate dehydrogenase (LDH) activity was measured by means of a conventional colorimetric assay using the LDH Cytotoxicity Detection kit (Roche Applied Science, Mannheim, Germany). Cells cultured in tissue culture polystyrene (TCPS) were used as a positive control and wells without cells were used as negative control. Absorbance was measured spectrophotometrically at 492 nm using a Power Wave Microplate Spectrophotometer (BioTek, Winooski, VT, USA). The percentage of cell survival was calculated as follows: cell survival (%) = [((experimental value − negative control)/(positive control − negative control)) × 100] [50,70].

### 4.5. In Vivo Model of Osseointegration

In vivo study was carried out in eighteen female adult New Zealand White (NZW) rabbits (Charles River, Saint Aubin les Elboeuf, France) aged six months, with an average body weight of 5 kg (range 4–6 kg). The study UIC-09432-2016 (23 July 2016) was approved by the Research Ethical Committee of the Facultad de Veterinaria of the Universidad Autónoma de Barcelona (UAB) and conducted according to the European Community guidelines for the care and use of laboratory animals (DE 86/609/CEE).

Rabbits were kept in individual cages and fed with commercial food and water ad libitum. The animals were anaesthetized by subcutaneous administration of Midazolam, Buprenorfine, and Medetomidine, followed by Alfaxalone for induction, without endotracheal intubation, and then maintained with Isoflurane and oxygen by mask during surgery.

The surgical procedure was performed under standard sterile conditions. After aseptic preparation of the surgical field, a skin incision was made in the lateral aspect of the distal femur. Once the subcutaneous fascia was dissected, metaphyseal bone was exposed and one monocortical hole was drilled in each femur with a 3 mm drill bit. The cylinder was introduced press-fit into the defect until it was flush with the cortex. The rabbits underwent bilateral surgery and received one sample in each femur, being samples of different type in each side of each animal. Rabbits were randomly divided into three groups. Six rabbits were operated on by placing a sample of type 1 in the right femur and a sample of type 3 in the left femur (group 1); six others carried a sample of type 2 in the right femur and a sample of type 3 in the contralateral femur (group 2); and a type 1 sample was implanted in the right femur and a sample of type 2 in the contralateral side in the other six rabbits (group 3). Thus, twelve specimens of each sample type were implanted.

The rabbits were euthanized under general anesthesia, 4 weeks after implantation by an intramuscular injection of sodium pentobarbiturate 200 mg/Kg (Dolethal, Vetoquinol, France). The segments of the femoral containing the implant were excised and immediately fixed in 10% formaldehyde solution to preserve tissue structure.

### 4.6. Preparation of Histological Samples

Samples were processed following the Donath method [43]. They were cut by a diamond saw EXAKT 310 CL (EXAKT Advanced Technologies GmbH, Norderstedt, Germany) to obtain bone sections that did not exceed 4 mm in thickness. Obtained pieces were immersed in formaldehyde solution for 48 h to assure bone tissue fixation. Samples were dehydrated by immersion in increasing concentrations of ethanol solutions (30%, 50%, 75%, 96%, and 100% *v*/*v*) for periods of 3 days each with constant stirring at 50 rpm. Once totally dehydrated, samples were embedded in methyl-methacrylate resin (Technovit 7200; Kulzer-Heraus GmbH, Wehrheim, Germany) using increasing concentration solutions of resin with ethanol (30%, 50%, 70%, and 100% *v*/*v*) for periods of 3 days each with constant stirring at 50 rpm. Samples at 100% resin solution were kept under vacuum conditions for 48 h to guarantee proper resin penetration into tissues. Then, samples were photo-polymerized in a light control unit (Histolux; Kulzer GmbH, Wehrheim, Germany) with external water cooling system. Samples were exposed to white light for 4 h, and 12 h to UV light, to obtain a solid transparent block to allow sample cutting and polishing, and avoiding any kind of fracture and/or pull-out of tissues during these procedures.

Resin blocks were cut into several cross-sections by the Exakt diamond saw with continuous water irrigation at a maximum rotation speed with minimum charge. Samples were polished by means of a grinding machine Exakt-400CS (EXAKT Advanced Technologies GmbH, Norderstedt, Germany), with parallelism control and using SiC progressively abrasive papers (600, 800, and 1200 grit). Finally, polished samples were carbon-coated using a sputtering technique for SEM examination under vacuum conditions. A nanometric carbon thin film deposited on the polished samples allowed for their correct analysis by SEM, and backscattered electrons detector (BSE) allowed the surface composition analysis required to identify and distinguish bone tissues from the titanium metal.

### 4.7. Histomorphometrical Characterization

Polished samples were observed individually using a FIB-SEM “Surface Scanning Electron Focused Ion Beam” (Carl Zeiss NTS GbmH, Oberkochen, Germany) equipment with a backscattered electron detector. Observation conditions were 15 kV of potential at 8 mm of working distance to achieve a resolution up to 1.1 nm in SEM-BSE mode.

SEM evaluation was performed by carrying out a sequential scan of polished transversal section surfaces of the cylindrical samples implanted. A total of nearly 150 SEM micrographs were merged by Image-J 1.46R software (ImageJ, NIH, Bethesda, MD, USA) for each sample to create a high-resolution and quality single picture at high magnification. Merged images were processed using Photoshop (Adobe Systems, Dublin, Ireland) and ImageJ in order to calculate the BIC, ROI, and total new bone formation.

**Figure 7:**
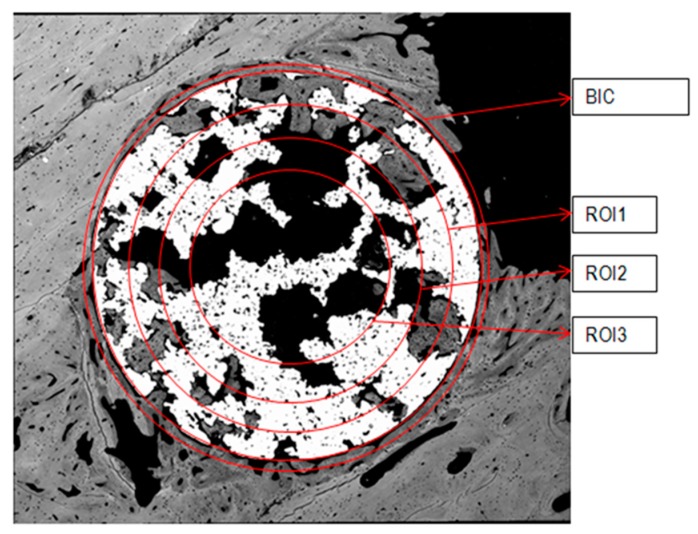
Scheme for assessment of BIC and ROI values.

BIC was used to quantify the percentage of bone tissue in intimate contact with the external perimeter implant. Newly formed bone around the implant and over its external surface corresponded to bone on-growth. New bone formed inside the porous structure was evaluated through ROIs quantification. Established ROIs consisted of four concentrically areas inside the implant surface from the perimeter to the center: ROI1, ROI2, ROI3, and center area [46]. ROI’s quantification was used to determine bone in-growth inside the porous scaffold (Figure 7). The total newly formed bone was calculated, taking into account the available space for bone growth throughout the interconnected porous scaffold.

## 5. Conclusions

After 4 weeks of implantation, no statistically significant differences were observed between the untreated scaffolds and the bioactivation treatments. However, comparing the two methods, the thermo-chemical treatment strategy seems to be the best option to enhance and accelerate bone tissue growth over the implant surface according to the BIC values achieved, while the peptide strategy yielded better trends in the inner core areas. We believe that further studies at longer time points are warranted to confirm these trends.

## Figures and Tables

**Table 1:** Characteristics of the original porous titanium structure, and with thermochemical treatment and peptide adhesion treatment: P is the average size of the pores, I is the interconnectivity of the porosity, and Ra is the roughness of the surface. References are the bibliographic references from other similar studies where other biomedical porous materials have tested in order to make a comparison with titanium foams.

Porous Material	P (μm)	I (%)	Ra (μm)	References
Ti porous	210 ± 9	57 ± 3	1.1 ± 0.1	
Ti porous thermochemical	208 ± 10	57 ± 2	1.1 ± 0.2	
Ti porous with peptides	210 ± 8	56 ± 3	1.3 ± 0.4	
Ta porous	370 ± 15	65 ± 5	1.4 ± 0.2	[47,48]
NiTi porous	350 ± 12	63 ± 6	1.1 ± 0.1	[49,50]

**Table 2:** Mechanical properties of the Ti foams tested in compression and fatigue. E is the Young’s modulus, σ_0_ is the yield stress, σ_max_ is the maximum strength, and ε is the strain to fracture obtained by compression tests. For the fatigue test, σ_f_ is the fatigue limit at 1 × 10^8^ cycles. The results have been compared with other biomedical porous materials and with the cancellous bone.

Porous Material	E (GPa)	σ_0_ (MPa)	σ_max_ (MPa)	ε (%)	σ_f_ (MPa)	References
Ti porous	0.61 ± 0.22	105.2 ± 10.8	170 ± 20.06	30.9 ± 4.6	16.4 ± 3.0	
Ti porous with thermochem.	0.66 ± 0.12	116.2 ± 9.7	177 ± 15.22	27.0 ± 4.6	15.4 ± 3.2	
Ti porous with peptides	0.63 ± 0.24	101.1 ± 9.8	165 ± 22.16	25.1 ± 4.6	13.5 ± 2.7	
Ta porous	1.15 ± 0.86	35.2 ± 0.8	71.2 ± 15.6	8.1 ± 1.8	7.5 ± 3.6	[47,48]
NiTi porous	1.21 ± 0.31	101.3 ± 14.3	142.5 ± 29.3	23.0 ± 4.1	13.2 ± 4.2	[49,50]
Cancellous bone	0.55 ± 0.48	15.2 ± 8.0	25.0 ± 8.1	7.1 ± 3.0		[48]
